# Concept of an Active Amplification Mechanism in the Infrared Organ of Pyrophilous *Melanophila* Beetles

**DOI:** 10.3389/fphys.2015.00391

**Published:** 2015-12-21

**Authors:** Erik S. Schneider, Anke Schmitz, Helmut Schmitz

**Affiliations:** ^1^Institute of Zoology, University of GrazGraz, Austria; ^2^Institute of Zoology, University of BonnBonn, Germany

**Keywords:** *Melanophila*, pyrophilous insect, infrared receptor, photomechanic receptor, fire detection, active amplification

## Abstract

Jewel beetles of the genus *Melanophila* possess a pair of metathoracic infrared (IR) organs. These organs are used for forest fire detection because *Melanophila* larvae can only develop in fire killed trees. Several reports in the literature and a modeling of a historic oil tank fire suggest that beetles may be able to detect large fires by means of their IR organs from distances of more than 100 km. In contrast, the highest sensitivity of the IR organs, so far determined by behavioral and physiological experiments, allows a detection of large fires from distances up to 12 km only. Sensitivity thresholds, however, have always been determined in non-flying beetles. Therefore, the complete micromechanical environment of the IR organs in flying beetles has not been taken into consideration. Because the so-called photomechanic sensilla housed in the IR organs respond bimodally to mechanical as well as to IR stimuli, it is proposed that flying beetles make use of muscular energy coupled out of the flight motor to considerably increase the sensitivity of their IR sensilla during intermittent search flight sequences. In a search flight the beetle performs signal scanning with wing beat frequency while the inputs of the IR organs on both body sides are compared. By this procedure the detection of weak IR signals could be possible even if the signals are hidden in the thermal noise. If this proposed mechanism really exists in *Melanophila* beetles, their IR organs could even compete with cooled IR quantum detectors. The theoretical concept of an active amplification mechanism in a photon receptor innervated by highly sensitive mechanoreceptors is presented in this article.

## Introduction

**Figure 1:**
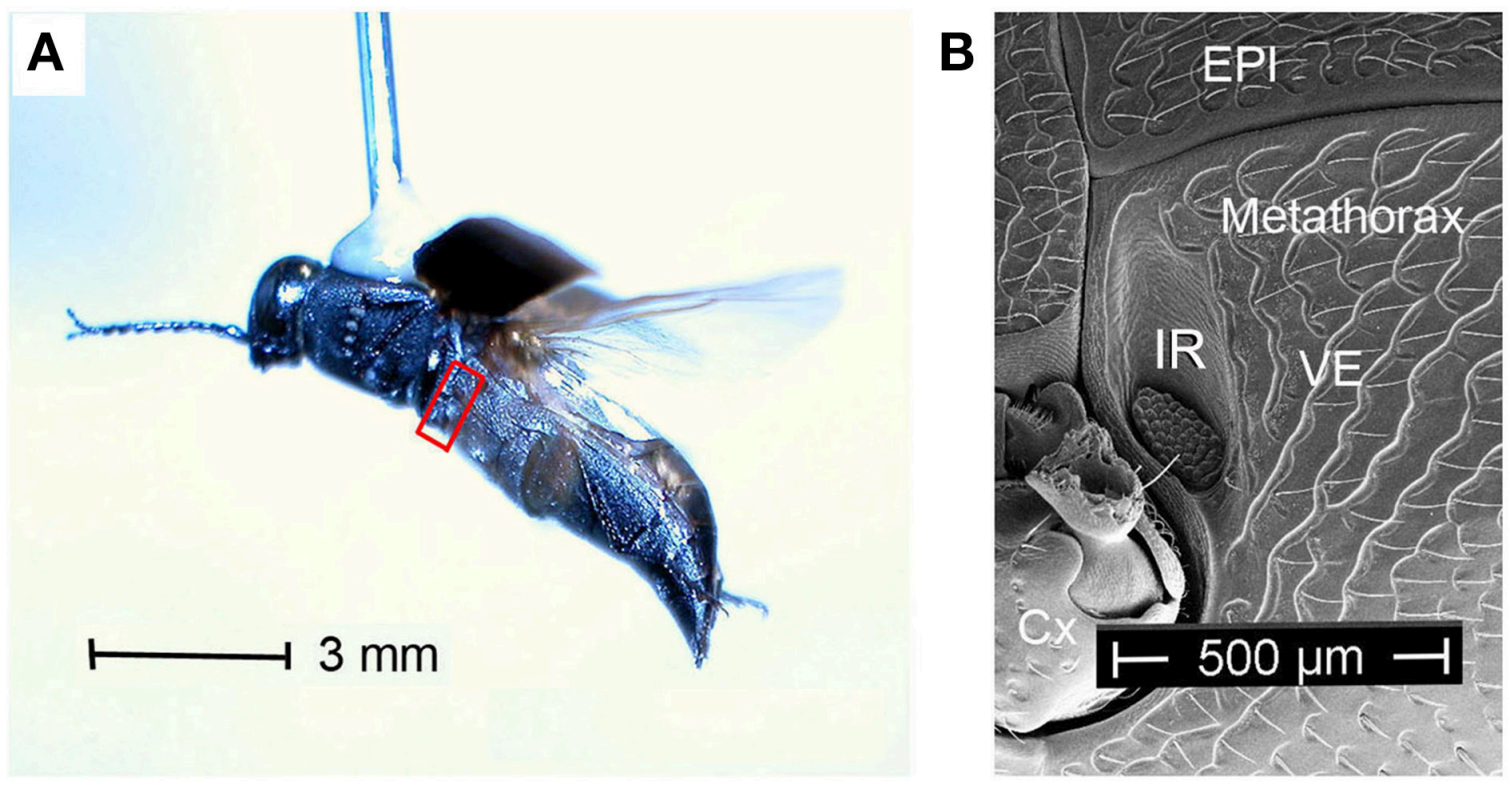
**(A)** *Melanophila cuspidata* dorsally glued to a needle flying in front of a wind tunnel. Red box encircles area shown in **(B)**. **(B)** Boundary between meso- and metathorax. Cx, coxa; EPI, episternite; VE, ventrite with IR organ (IR) close to the boundary to the mesothorax.

**Figure 2:**
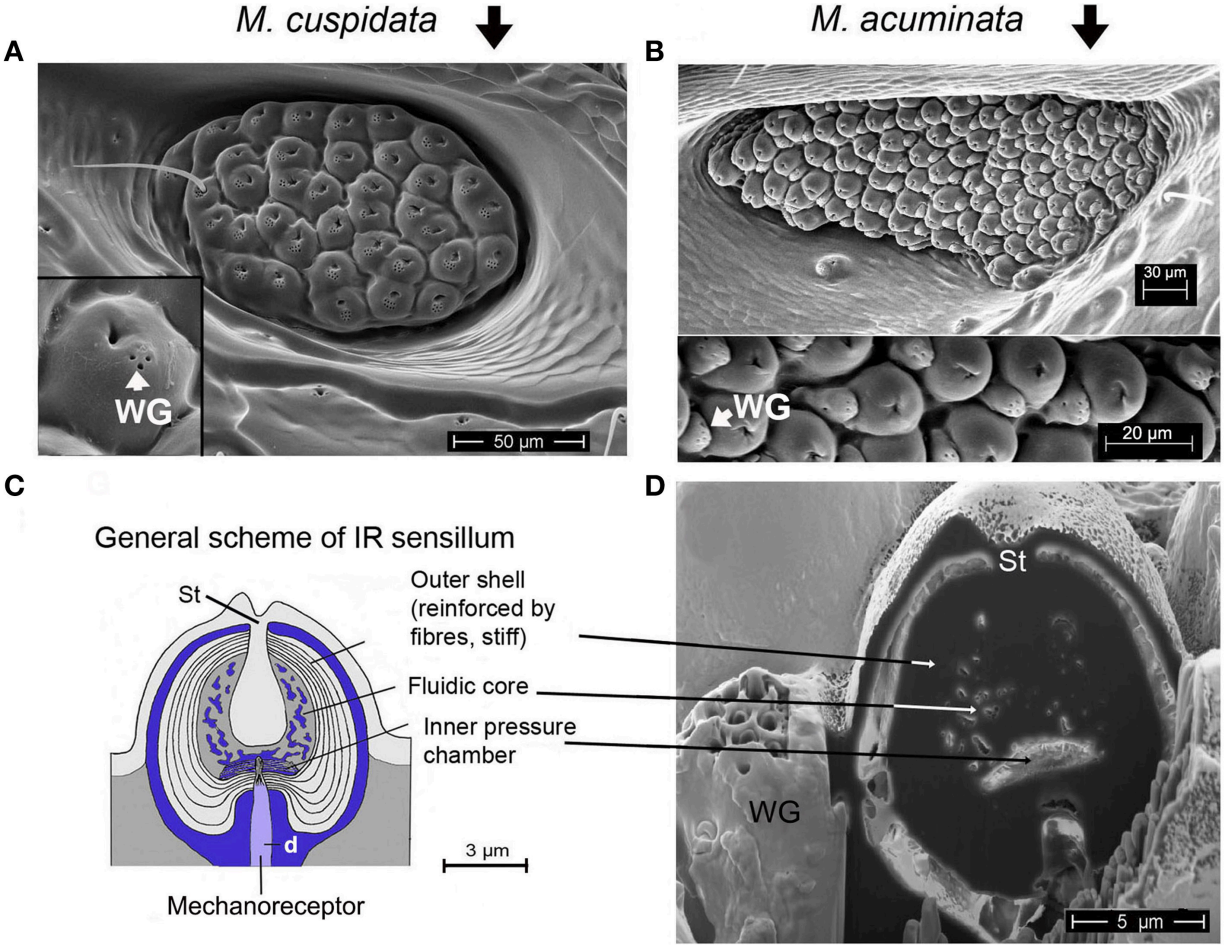
**(A)** IR organ of *Melanophila cuspidata*. About 40 sensilla are inside the pit organ. Sensilla belong to a more basic type because wax glands (WG) are still intergrated into the domes of the IR sensilla (inset shows single sensillum). On the left a not fully differentiated sensillum can be seen which still has a bristle. **(B)** IR organ of *Melanophila acuminata*. The pit organ contains more than 80 sensilla of a more specialized type (wax glands clearly separated from the domes, see lower part of the image). Image modified after Schmitz et al. (2007). **(C)** General scheme of a *Melanophila* sensillum. d, dendrite; St, stalk. Image modified after Schmitz et al. (2007). **(D)** Air dried sensillum opened up in the center with a focused ion beam (FIB). WG, wax gland; St, stalk.

With 13 recent species jewel beetles of the genus *Melanophila* mainly can be found in the boreal and temperate forests of the holarctic zone (Bellamy, 2008). According to the current state of knowledge, males and females approach forest fires because their larvae can only develop in wood of freshly burnt trees (Linsley, 1943; Apel, 1988, 1989, 1991). As an adaptation to the pyrophilous way of life the 1 cm long black beetles (Figure 1A) are equipped with special antennal smoke receptors (Schütz et al., 1999) and one pair of metathoracic IR organs (Evans, 1964; Vondran et al., 1995; Schmitz et al., 1997). An IR organ consists of a little array of dome-shaped sensilla which is situated at the bottom of a little pit (Figures 1B, 2A,B). The inner spherule of each sensillum is innervated by a ciliary mechanosensitive cell (Figure 2C; Vondran et al., 1995; Schmitz et al., 2007). Thus, the IR sensilla do not only respond to IR radiation but also to mechanical stimuli. A bimodality has already been demonstrated by electrophysiological experiments: single sensilla respond to weak vibratory stimuli with distinct action potentials (see Figure 3C in Schmitz and Bleckmann, 1998).

There are several hints in the literature that beetles are not only able to detect forest fires but also fires and heat sources of anthropogenic origin from distances of 30 km, 60 km, and even more than 100 km (Van Dyke, 1926; Linsley, 1943; Linsley and Hurd, 1957). Currently it seems not very likely that beetles use the smell of smoke to detect fires from greater distances. It could not be demonstrated that *Melanophila* beetles could be lured by the smell of smoke (Evans, 1964) or that beetles resting at temperatures of 25°C could be aroused by smoke (unpublished data). A recent study, however, shows that beetles can be attracted by certain volatiles emitted by burning or smoldering wood (Paczkowski et al., 2013). In the study crawling beetles were tested in a two arm olfactometer at a temperature of 30°C. No information about the sex and mating state of the beetles is provided in this study. So these data are more likely suited to show that beetles (e.g., mated females?), once having landed on a burnt tree, can detect a suitable spot for oviposition by olfactory cues. Evaluations of satellite images very often yielded the result that the large smoke plume from a forest fire initially is driven away from the fire by the wind in a narrow angle over distances of many kilometers and finally gradually ascends to higher altitudes. So only beetles inside the smoke plume have a chance to become aware of the fire by olfactory cues. In contrast, beetles that are already close to the fire but outside the smoke plume most probably can see the plume but are not able to smell the smoke. Also the light of the flames—generally only visible at night—may not play an important role, because *Melanophila* beetles, as nearly all jewel beetles, are diurnal (Evans et al., 2007).

All threshold sensitivities published so far were measured in non-flying beetles (highest sensitivity 60 μW/cm^2^, see Table 1). Theoretical calculations, however, show that these sensitivities only allow a detection of a large fire from a distance of about 12 km (Evans, 1966; Schmitz and Bleckmann, 1998).

**Table 1:** **Sensitivity thresholds of biological and technical IR sensors**.

	**Threshold**	**Source/Explanation**
*Melanophila* Behavioral experiments	60 μW/cm^2^	Evans, *Ecology* (1966); Determined by behavioral experiments with non-flying beetles
Pyroelectric sensor (room temperature) (DIAS 256 LTI)	8.4 μW/cm^2^	Sensorarray DIAS Infrared, Dresden
Rattlesnake *Crotalus atrox*	3.3 μW/cm^2^	Ebert and Westhoff, *JCP A* (2006)
Microbolometer (room temperature)	2.3 μW/cm^2^	Sensorarray Information provided by Dr. N. Hess, DIAS Infrared GmbH, Dresden. Compiled after: annual SPIE-Conferences “Infrared Technology,” Orlando, USA, 1999–2011
Cooled quantum detector (AIM HgCdTe)	79 nW/cm^2^	Sensorarray AIM Infrarotmodule GmbH, Heilbronn AIM Data Sheet 2nd-3rd-Generation
*Melanophila* Coalinga-Simulation	40 nW/cm^2^	Schmitz and Bousack, *PLoS ONE* (2012)
Cryogenic cooled quantum detector (Hamamatsu P5274 Serie)	250 pW/cm^2^	High sensitivity single element quantum detector Hamamatsu MCT photoconductive detectors Data sheet P2748/P5274 series

The results of a simulation of a huge man-made fire provide further evidence that *Melanophila* beetles might be able to detect IR radiation emitted by remote fires from much larger distances (Schmitz and Bousack, 2012). In this study a big oil-tank fire was modeled that burnt in 1925 for 3 days in Coalinga (California) and attracted “untold numbers” of *Melanophila consputa*. This event has also been documented in the entomological literature (Van Dyke, 1926). The site of the fire in the woodless Central Valley of California suggests that most beetles detected the fire by IR radiation from distances of 130 km. This would imply a threshold sensitivity of only 40 nW/cm^2^ (Table 1), corresponding to an energy at a single sensillum of 1.3 × 10^−17^ J. If the threshold of the *Melanophila* IR organ should really be in this range, the biological IR receptor would be two orders of magnitude more sensitive than all current uncooled technical IR sensors and would be able to compete with much more expensive cooled quantum detectors (cf. Table 1).

However, a sensitivity three orders of magnitude higher than the highest sensitivity ever published (Table 1) is only explainable by active amplification mechanisms. Until now, active amplification of very weak input signals has only be reported in the context of hearing: in the cochlear amplifier in the inner ear of vertebrates (Hudspeth, 1989, 1997; Gillespie and Müller, 2009), in antennal ears of mosquitoes and the fly *Drosophila* (Göpfert and Robert, 2001, 2003; Göpfert et al., 2005, 2006; Mhatre, 2015) and recently discovered also in the tympanal ears of a tree cricket (Mhatre and Robert, 2013). An amplification of 1000-fold can be achieved by electromotile outer hair cells in the mammalian cochlea (Robles and Ruggero, 2001; Ashmore et al., 2010). Like hair cells and scolopidia in vertebrate and insect ears, respectively, the mechanosensitive sensory cells that innervate the IR sensilla in *Melanophila* are ciliary mechanoreceptors. Therefore, the search for active amplification mechanisms appears highly challenging.

## Morphological prerequisites

**Figure 3:**
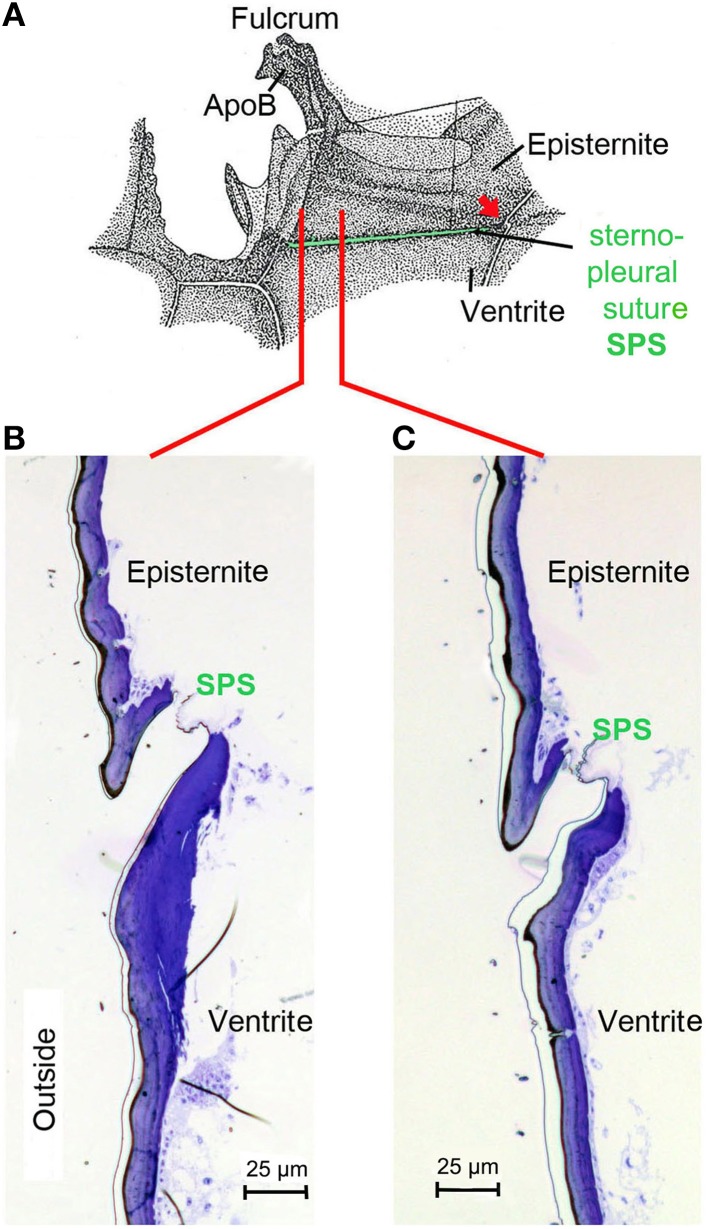
**(A)** Morphology of the pleural region of the metathorax in beetles shown here for the rhinoceros beetle *Oryctes rhinoceros* (after Darwin and Pringle, 1959). The episternite with the fulcrum and the apodem of the basalar muscle (ApoB) are connected by a posterior hinge (red arrow) to the ventrite. In the region of the sternopleural suture (SPS, green) a hinge membrane allows movement of the episternite against the ventrite (Pringle, 1957). **(B)** Dorsoventrally oriented section through episternite and ventrite in the anterior region (indicated by red line in **A**) in the buprestid beetle *Chrysobothris solieri*. **(C)** Corresponding section about 150 μm in posterior direction.

**Figure 4:**
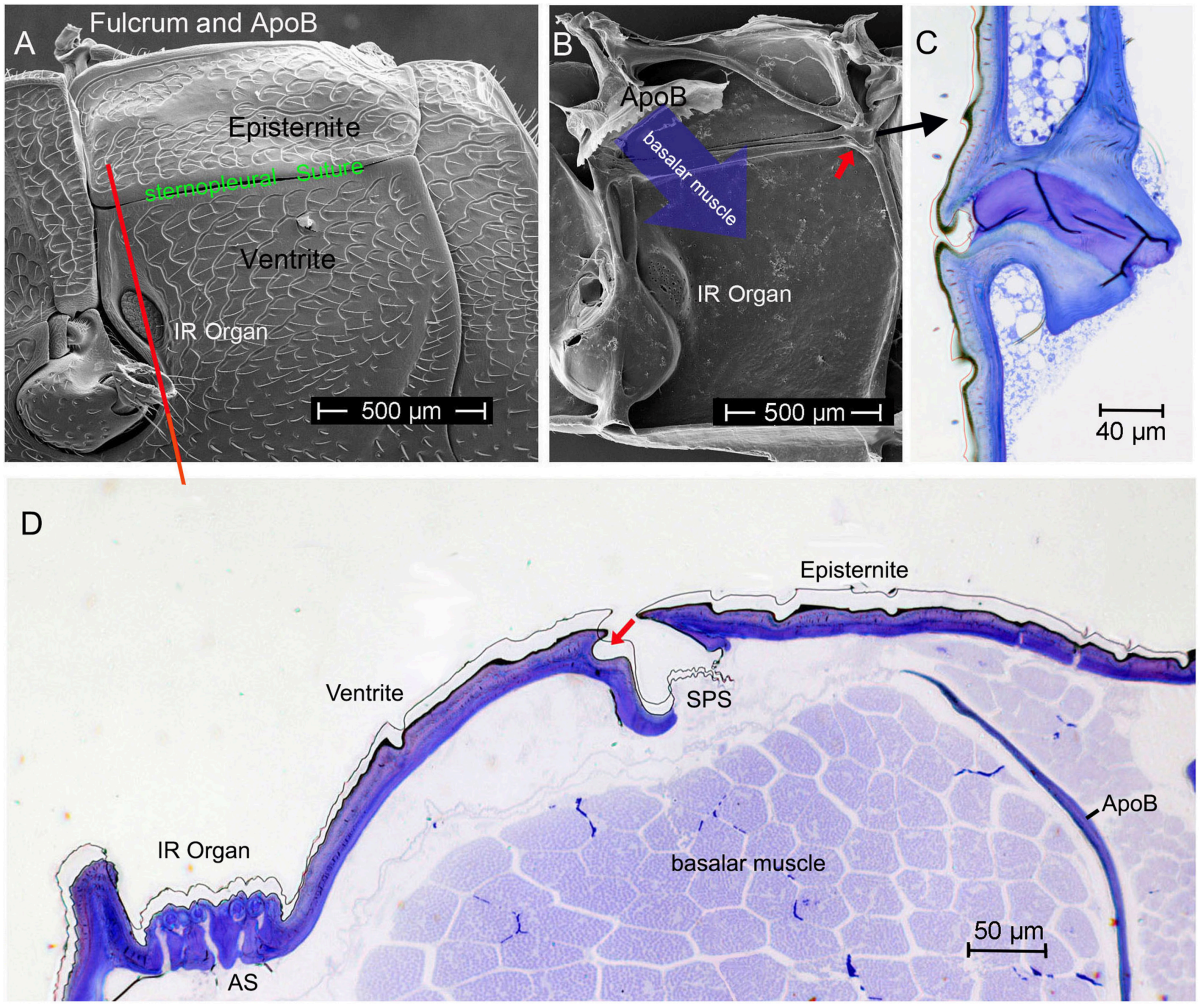
**(A)** Outer and **(B)** inner view of the pleural metathorax of *Melanophila cuspidata*. The ventral anterior region of the episternite is developed as impact edge which can be beaten in a furrow of the ventrite. In **(B)** the apodeme of the basalar muscle (ApoB) is shown which is connected to the fulcrum (tissue removed). Red arrow points to posterior hinge shown in **(C)**. **(C)** Section through hinge in dorso-ventral direction, outside on the left). **(D)** Dorso-ventral section through the pleural metathorax (indicated in **A** by red line). By contraction of the basalar muscle the edge of the episternite is beaten in the dorsal furrow of the ventrite (red arrow). Vibrations caused by the beats most probably are conducted to the IR sensilla. AS, air sac below IR organ; SPS, sternopleural suture.

Starting point for the development of the concept was the consideration that the IR organs are situated on the metathorax just below the wing hinges (fulcra) of the alae (Figures 1A, 4A,B). Thus, the IR organs are located at a site strongly subjected to vibrations during flight. Additionally, a detail so far not understood was considered: the sphere of a sensillum is attached by a little stalk to the outer cuticular dome (Figures 2C,D). These stalks are missing in the photomechanic IR sensilla of pyrophilous *Aradus* bugs which are quite similar to the *Melanophila* IR sensilla but are not enclosed in a pit organ (Schmitz et al., 2010). By this constellation, vibrations of the spheres in flying *Melanophila* beetles can be proposed to affect the receptor potentials of the mechanosensory cells. To realize genuine amplification, however, a mechanism has to be implemented permitting a precise regulation of the depolarization amplitude. The morphological prerequisites for such a regulation mechanism were found in the two hitherto investigated species *Melanophila acuminata* and *M. cuspidata*. The structural preadaptation for the evolution of an adjustable beat mechanism most probably was a special feature of the metathoracic pleural region in beetles: the sternopleural suture (SPS, Figures 3A–C). Episternite and ventrite are tightly connected only by a posterior hinge and thus can be moved against each other—especially at their anterior edges (Pringle, 1957). Amongst other purposes this mechanism mainly serves for lifting the fulcrum during flight. When the basalar muscle contracts, the episternite is pulled against the ventrite and the leading edge of the wing is pronated during the downstroke (Darwin and Pringle, 1959). The opposing edges of the episternite and the ventrite are formed in a manner so that the episternite can glide over the ventrite (as in the non IR-sensitive jewel beetle *Chrysobothris solieri*, closely related to the *Melanophila*-species, Figures 3B,C).

**Figure 5:**
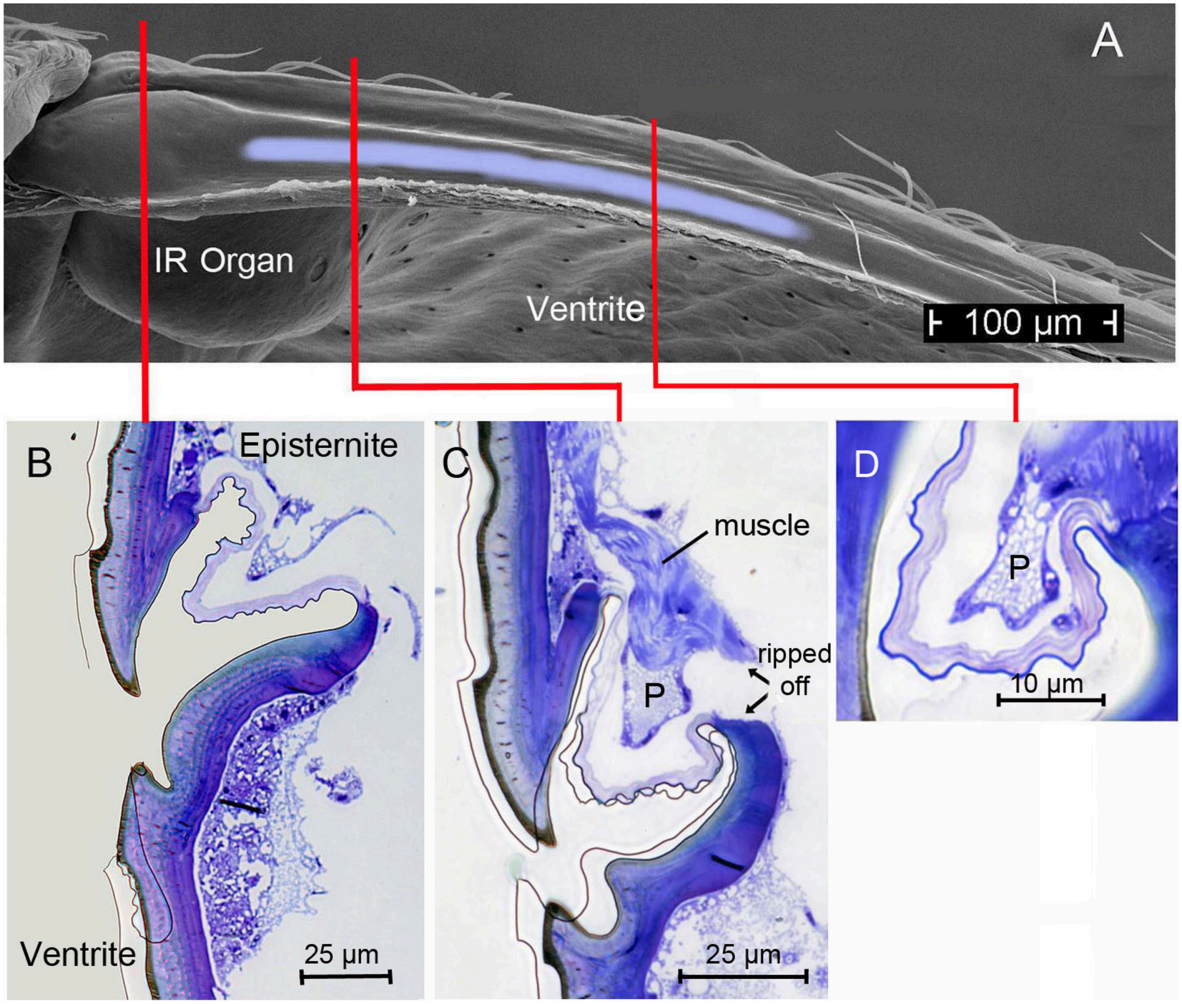
**(A)** Top view (SEM micrograph) onto the furrow at the dorsal edge of the ventrite in *Melanophila acuminata* (all tissue removed). Light-blue area indicates region in which the proposed damping cushion (pad) can be brought into contact with the ventrite. **(B)** Section through region marked with red line in **(A)** showing the beating edge of the episternite and the narrow furrow in the ventrite. Same orientation as in Figure 4A. **(C)** Section through region marked in **(A)** with red line: here the damping pad (P) with its musculature is shown. **(D)** Section through the pad about 150 μm posteriorly. Pad becomes smaller toward its posterior end. After about 300 μm the pad is not longer present.

It turned out that in IR-sensitive *Melanophila*-species especially the morphology of the opposing anterior edges of episternite and ventrite has been modified. The slim ventral edge of the episternite can be beaten in a trench on the dorsal edge of the ventrite, thus a kind of impact edge is realized (Figures 4D, 5A–C). According to the current conception beating of the two sclerites against one another is accomplished by contractions of the basalar muscle, which extends from the dorsal apodeme (ApoB; shown in Figure 4B) posteriorly to the basal region of the ventrite. By regulating the power of the muscle contractions during a search flight sequence (see below), the vibrations of the spheres caused by the proposed mechanism can be controlled. Therefore, also the magnitude of receptor depolarizations could be regulated.

In this context it is of great importance that the IR sensilla are innervated by ciliary mechanoreceptors. Specialized arthropod mechanoreceptors innervated by ciliary mechanosensory cells are the most sensitive receptors known. This could be shown for trichobothria in spiders, where energies of 1.5 × 10^−19^ J to 2.5 × 10^−20^ J are still sufficient for a suprathreshold stimulation of the receptors (Humphrey et al., 2003; Barth, 2004) and also for filiform hairs in insects (Thurm, 1982). Filiform hairs in crickets serving for detection of faint airflows can already generate an action potential if energies are still in the range of k_B_T (k_B_: Boltzmann constant; T: temperature), i.e., about 4 × 10^−21^ J (Shimozawa et al., 2003).

At the threshold, these ultrasensitive mechanoreceptors already work within the range of thermal noise of Brownian molecular motion and therewith close to the physical limit (Barth, 2004). It can be concluded that for a subthreshold depolarization of the mechanosensitive sensory cell innervating the IR sensilla most probably vibration amplitudes of the spheres of less than one nanometer are already sufficient.

In the two *Melanophila* species investigated so far, *M. acuminata* and *M. cuspidata*, morphological differences were found. It appears that the IR detection system (i.e., IR-organ plus the structures used for the proposed beat mechanism) in *M. cuspidata* is more ancient and relatively simple: IR organs contain less sensilla into which the wax glands are fully integrated (Figure 2A). So an unrestrained vibration of the sphere most probably may be somewhat hampered and less precise. An explanation could be that *M. cuspidata* is distributed in the Mediterranean region where fires are more frequent than in northern Europe (San-Miguel and Camia, 2009). Thus, the necessity to detect fires also from very large distances seems not to be predominant. In contrast, *M. acuminata* is distributed in the boreal forests of the northern hemisphere (in Europe northern distribution up to Fennoscandia; Horion, 1955), where forest fires are less frequent. Accordingly, a higher evolutionary pressure with respect to the sensitivity of the IR organs can be proposed. In *M. acuminata*, the IR organs contain significantly more IR sensilla from which wax glands are clearly separated (Figures 2B–D). It can be concluded that unobstructed vibrations of the spheres are possibles. Furthermore, an additional damping system obviously has developed. This system may allow a precise “fine tuning” of the depolarizations of the IR sensilla caused by the beat mechanism. By a system of at least two muscles of hitherto unknown origin a damping cushion of about 300 μm lengths can be brought down into the inner trench of the ventrite (Figures 5A–D). Thus, at a given contraction power of the basalar muscle a very precise adjustment of the beat intensity and consequently of the evoked pre-depolarizations could be adjusted. Despite intense search in two further specimens such a damping cushion could not be found in the Mediterranean *M. cuspidata* (cf. Figure 4).

## How it could work

**Figure 6:**
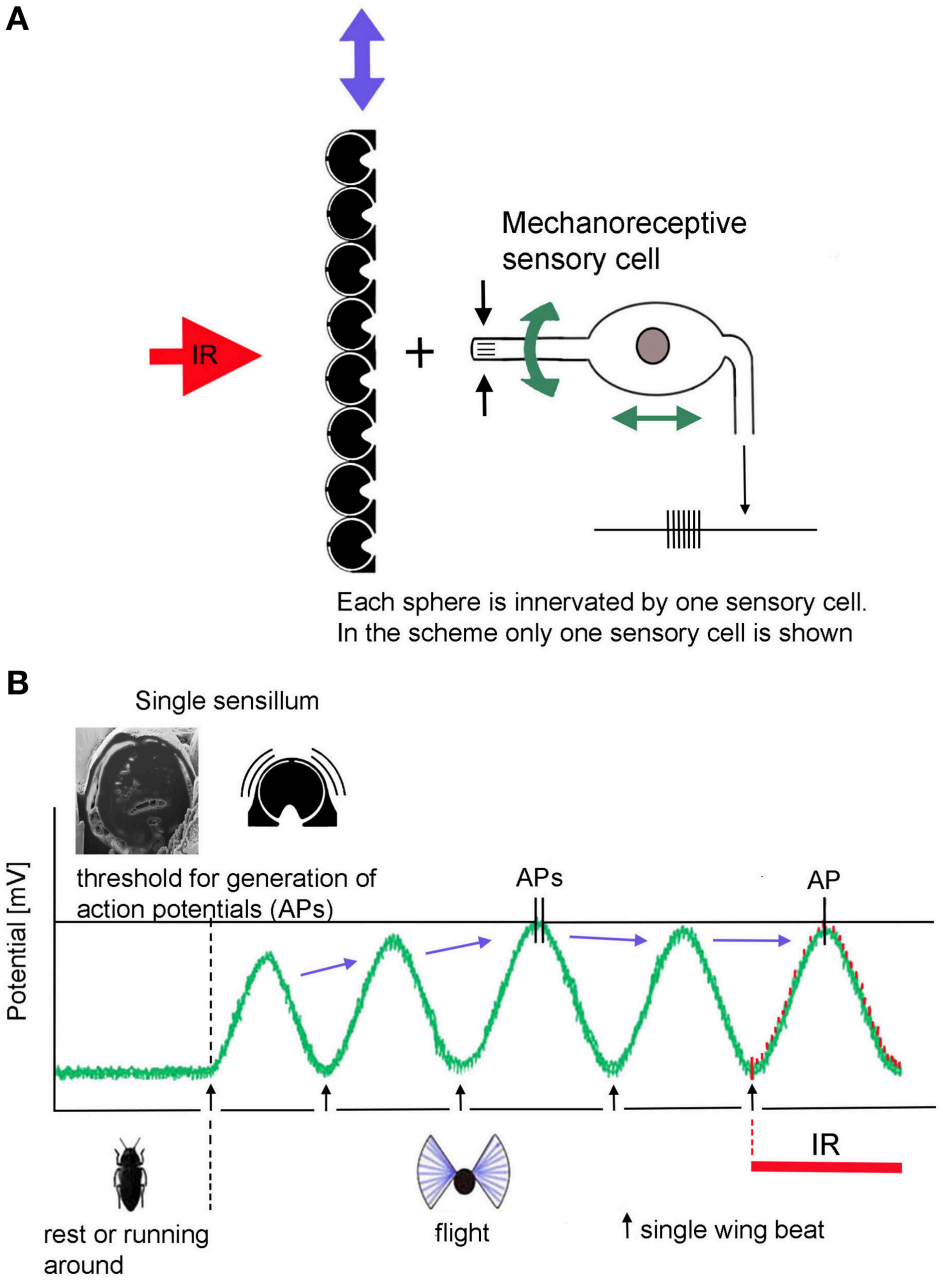
**Schematic depiction of the proposed beating mechanism**. **(A)** Coupling in muscular energy (blue double arrows) from the flight motor results in vibration of the cuticular spheres. This in turn causes the stimulation of the mechanoreceptive sensory cells by which the spheres are innervated. By sensory feedback the sensory cells can be systematically depolarized with wing beat frequency. In contrast, energy for active amplification in hearing organs is generated by molecular motors of the sensory cells itself (green double arrows at the sensory cells). **(B)** In non-flying beetles the receptor potential of the IR sensilla is set at about −70 mV. Slight fluctuations are caused by thermal and mechanical noise. When the beetles take off, IR sensilla become depolarized by the beating mechanism: (1) by increasing beat intensity, the beetle is able to release action potentials in the absence of IR radiation. (2) by tuning the beat intensity and—in *Melanophila acuminate*—by additional fine tuning of the damping system, depolarization can be adjusted in a way that the generator potentials of most sensilla remain just below the threshold. (3) in turn IR radiation superimposed to the thermal noise at the peaks of the receptor potentials causes the generation of (additional) action potentials in exposed sensilla.

According to the present idea how *Melanophila* beetles may be able to become aware of a fire from large distances, beetles use a combination of visual cues (view of a big cloud against the horizon) and IR radiation. To make sure that a smoke plume and not a cloud bank is approached over distances of many kilometers a zone of IR emission has to exist at the base of the cloud above tree top level. IR sensitive *Melanophila* beetles, therefore, will conduct search flights for fire detection. While doing so, beetles especially examine potential smoke plumes in view of additional IR emission. By the beat mechanism a cyclic depolarization of the IR sensilla with wing beat frequency appears possible (Figures 6A,B). In beetles, the basalar muscle serves as a well-developed direct wing depressor, which, together with the indirect dorso-longitudinal muscles (main muscle for propulsion), is used for wing downstroke. In principle the basalar muscle can be classified as a steering muscle (Nachtigall, 2003). By adjusting the contraction power of the basalar muscle, the wing inclination angle during the downstroke of the wing (pronation) and in this way propulsion and buoyancy is adjusted. During a supposed search flight sequence, which may last for a few seconds only, the beetles could tune the contractions of the basalar muscles exactly so that the peak amplitudes of the oscillatory receptor potential almost reach the spike-triggering threshold. At the same time a slight reduction in propulsion and buoyancy would not be disadvantageous. To tune the IR organs to maximal sensitivity in anticipation of arriving IR radiation, the beetles should be able to adjust the intensity of the beat mechanism and therewith the probability of impulse generation by sensory feedback. As a result only a certain, most probably very low, percentage of sensilla in both organs generate action potentials.

With respect to symmetry the inputs of both IR organs could be permanently compared by central comparator neurons. Such central units enable acoustically communicating insects to approach, e.g., a sound source by paired hearing organs (von Helversen and von Helversen, 1995; Stumpner and von Helversen, 2001). A mechanical prestimulation of only a few sensilla in the *Melanophila* IR organ could be explainable by the fact that the sensilla show minute differences in their dimensions (cf. Figures 2A,B). In case of an oscillatory mechanical stimulation with constant intensity some sensilla will already generate first action potentials whereas most others will remain just below the threshold. At the peak of a given subthreshold depolarization additional IR radiation will slightly increase the height of the amplitude (Figure 6B). This will result in a few more spiking sensilla in the organ exposed to IR radiation. The asymmetry in the inputs of both organs immediately could be detected by comparator neurons. Therewith the beetle gets information about the spatial direction from which IR radiation arrives. This information could be combined with visual cues (e.g., a smoke plume) and the beetle should be able to directly approach a fire.

Based on theoretical considerations it seems essential that the vibrations of the spheres caused by the proposed beat mechanism have to be strongly damped. By appropriate damping an uncontrolled soaring up of the system can be suppressed and it can be ensured that the spheres all are in a defined initial state before the next impact impulse arrives. Ideally a creeping case (i.e., damping so strong that no oscillation can arise) or at least an aperiodic limit case (i.e., strong damping ensures oscillation of the sphere with only one zero crossing) has to be proposed. In this way beat impulses of constant intensity will always cause monotonic depolarization amplitudes. Most probably damping is realized by a slender margin of fluid surrounding each sphere (Figure 2C). This margin with a thickness of about 0.3 μm consists of the apical extensions of the two outer enveloping cells (Vondran et al., 1995). By this specific feature a fluidic damping system is build. Subthreshold depolarizations of the receptors most probably are already evoked by sub-nanometer vibrations of the spheres. The necessary small scale dislocation of water is allowed by compensatory air sacs below the IR organ (Figure 4D, AS).

Provided that the cuticular apparatus (i.e., mainly the spheres) is able to convert the energies of absorbed IR photons effectively into mechanical energy (so-called photomechanic mechanism of IR reception, Schmitz and Bleckmann, 1998) it could be possible that the sensitivity threshold of a *Melanophila* IR organ is about thousand fold lower (analogous to the mammalian cochlear amplifier) than the hitherto published lowest threshold of 60 μW/cm^2^.

## Closing remarks

Among biological IR sensory systems it is a unique feature of *Melanophila* beetles that IR sensilla serve as photon receptors although they are innervated by mechanosensitive neurons. The cuticular apparatus absorbs incoming IR radiation and transforms photon energy into a micromechanical event measured by a dedicated mechanoreceptor. In principle this constellation provides the possibility of active amplification of faint mechanical input signals. As mentioned in the Introduction active amplification has been shown in the context of hearing: for the hair cells in the cochlear amplifier in vertebrates (Hudspeth, 1997; LeMasurier and Gillespie, 2005; Fettiplace and Hackney, 2006; Ashmore et al., 2010) but also for the chordotonal organs in the ears of certain flies (Göpfert and Robert, 2001; Göpfert et al., 2005; Nadrowski et al., 2008) and a tree cricket (Mhatre and Robert, 2013). In hearing, however, the energy required for amplification is expended by the sensory cells themselves whereas in the proposed active IR receptors in *Melanophila* beetles the energy originates from the flight motor. Thus, in principle, the proposed mechanism to achieve a high sensitivity in a receptor for electromagnetic radiation is new. We further suggest that three different mechanisms are involved: (i) as proposed active signal amplification, (ii) active sensing which means that activity of the sensor system already starts in anticipation of a stimulus (Nelson and MacIver, 2006), and (iii) stochastic resonance: noise—in this case self-generated—is used for better signal detection (Harmer et al., 2002; Moss et al., 2004; McDonnell and Abbott, 2009).

The proposed beat mechanism, however, shows some marked differences compared to the mechanisms mentioned. No energy used for target analysis is emitted into the surrounding (difference to conventional *active sensing*), muscular energy is used for signal amplification (fundamental difference to the cellular molecular motors of the sensory cells in ears) and the crucial part of the “noise” is produced by self-generated oscillations. For the purpose of ultrasensitive stimulus detection the probability of action potential generation can be adjusted by altering the overall noise amplitude (difference to stochastic resonance that only works at an optimal noise intensity, which can hardly be influenced by the sensor system).

If the proposed high sensitivity of the IR organ could be demonstrated, the biological IR sensor would advance into the sensitivity gap currently existing between relatively cheap uncooled thermal IR sensors and expensive cooled quantum detectors requiring much more effort during operation and also more costly service (see Table 1). Thus, the demonstration of the postulated amplification mechanism would also be of technical interest for the development of new active IR sensors.

### Conflict of interest statement

The authors declare that the research was conducted in the absence of any commercial or financial relationships that could be construed as a potential conflict of interest.
